# Supplementary material for: Fauna of the Kemp Caldera and its upper bathyal hydrothermal vents (South Sandwich Arc, Antarctica)
Source: R Soc Open Sci. 2019 Nov 20;6(11):191501. doi: 10.1098/rsos.191501 (PMC6894572; doi:10.1098/rsos.191501)
Supplement: Table S1 [file rsos191501supp1.docx]

Supplementary Table 1

Fauna of the Kemp Caldera and its upper bathyal hydrothermal vents (South Sandwich Arc, Antarctica)

Katrin Linse, Jonathan Copley, Douglas P. Connelly, Robert D. Larter, David A. Pearce, Nick V.C. Polunin, Alex D. Rogers, Chong Chen, Andrew Clarke, Adrian G. Glover, Alastair G.C. Graham, Veerle A.I. Huvenne, Leigh Marsh, William D.K. Reid, C. Nicolai Roterman, Christopher J. Sweeting, Katrin Zwirglmaier, Paul A. Tyler

Summary of ROV *Isis* deployments in the Kemp Caldera. Tasks, collections and other: Ex = experiment deployment, F = Fauna collected by ROV claw, N = Niskin, S = Suction sampler, Sw = Swath bathymetry, Ti = Titanium sampler, Tr = Trap, T-L = temperature lance, VM = Video mosaic
