# Supplementary material for: Fauna of the Kemp Caldera and its upper bathyal hydrothermal vents (South Sandwich Arc, Antarctica)
Source: R Soc Open Sci. 2019 Nov 20;6(11):191501. doi: 10.1098/rsos.191501 (PMC6894572; doi:10.1098/rsos.191501)
Supplement: Table S2 [file rsos191501supp2.docx]

Supplementary Table 2

Fauna of the Kemp Caldera and its upper bathyal hydrothermal vents (South Sandwich Arc, Antarctica)

Katrin Linse, Jonathan Copley, Douglas P. Connelly, Robert D. Larter, David A. Pearce, Nick V.C. Polunin, Alex D. Rogers, Chong Chen, Andrew Clarke, Adrian G. Glover, Alastair G.C. Graham, Veerle A.I. Huvenne, Leigh Marsh, William D.K. Reid, C. Nicolai Roterman, Christopher J. Sweeting, Katrin Zwirglmaier, Paul A. Tyler

Presence/absence data for vent taxa compiled from published literature for the Kemp Caldera and 15 well-studied vent fields in neighbouring oceanic regions: the Southern Ocean (E2 and E9 vent fields), the Indian Ocean (Longqi, Duanqiao, Tiancheng, Kairei, Edmond, Solitaire, and Dodo fields), and Mid-Atlantic Ridge (Lucky Strike, Rainbow, Broken Spur, TAG, Snake Pit, Ashadze-1, and Logatchev fields), updating the dataset previously published by Copley et al. (2016) with subsequently published records of additional sites and taxa (Zhang et al., 2017; Chen et al., 2018; Watanabe et al., 2018; Zhou et al., 2018).

Vent field abbreviations: Ashadze-1 = A1, Broken Spur = BS, Duanqio = Du, Edmond = Ed, Kairei = Ka, Kemp Caldera = KC, Logatchev = Log, Longqi = Lon, Lucky Strike = LS, Rainbow = Ra, Snake Pit = SP, Solitaire = So, Tiancheng = Ti
